# Allostatic Load, Educational Attainment, and Risk of Cancer Mortality Among US Men

**DOI:** 10.1001/jamanetworkopen.2024.49855

**Published:** 2024-12-10

**Authors:** Cynthia Li, Sydney P. Howard, Charles R. Rogers, Sydney Andrzejak, Keon L. Gilbert, Keith J. Watts, Malcolm S. Bevel, Myles D. Moody, Marvin E. Langston, Judah V. Doty, Adetunji T. Toriola, Darwin Conwell, Justin X. Moore

**Affiliations:** 1 Center for Health Equity Transformation, Department of Behavioral Science, University of Kentucky, Lexington; 2 Medical College of Georgia, Augusta University, Augusta; 3 Divison of Epidemiology & Social Sciences, Institute for Health and Equity, Medical College of Wisconsin, Milwaukee; 4 Mercer University School of Medicine, Macon, Georgia; 5 Behavioral Science and Health Equity, College for Public Health and Social Justice, Saint Louis University, Saint Louis, Missouri; 6 College of Social Work, University of Kentucky, Lexington; 7 Georgia Cancer Center, Augusta University, Augusta; 8 Department of Sociology, College of Arts and Sciences, University of Alabama at Birmingham, Birmingham; 9 Department of Epidemiology, Stanford University, Stanford, California; 10 Division of Public Health Sciences, Department of Surgery and Siteman Cancer Center, Washington University School of Medicine, Saint Louis, Missouri; 11 Department of Internal Medicine, College of Medicine, University of Kentucky, Lexington

## Abstract

**Importance:**

Health disparities among racial and ethnic minoritized populations, particularly for cancer mortality rates, remain a major public health concern. Men from underrepresented backgrounds (Black and Hispanic men, specifically) face the pervasive effects of discrimination in their daily lives, which also contribute to the complex associations among allostatic load (a marker of chronic stress), educational opportunities, and elevated risks of cancer mortality.

**Objective:**

To elucidate the associations among educational attainment, allostatic load, and cancer mortality risk among men.

**Design, Setting, and Participants:**

This is a retrospective cohort analysis of data from the National Health and Nutrition Examination Survey, a nationally representative sample of approximately 5000 people across the US, from 1988 to 2010 linked with data from the National Death Index, which served as follow-up data for the cohort and was available through December 31, 2019. Participants included men aged 18 years and older. Data were analyzed from June to October 2024.

**Exposure:**

Allostatic load data were stratified by educational attainment levels, categorized as (1) less than high school education and (2) high school graduate and above. Allostatic load score was calculated as the sum of total abnormal biomarkers and health measures (9 total). Participants were considered to have high allostatic load if their score was 3 or more.

**Main Outcomes and Measures:**

The primary outcome was cancer death. Weighted Cox proportional hazards models were fitted to estimate adjusted hazard ratios (HRs) of cancer death between educational attainment and allostatic load (adjusted for age, income, and smoking status).

**Results:**

Among all 20 529 men (mean [SE] age, 41.00 [0.22] years), those with high AL and less than high school educational attainment had a greater than 4-fold increased risk of cancer mortality (unadjusted HR, 4.71; 95% CI, 3.36-6.60) compared with those with low allostatic load and a college degree or higher. Similarly, both Black men (HR, 4.19; 95% CI, 2.09-8.40) and White men (HR, 5.77; 95% CI, 4.06-8.20) with high allostatic load and less than high school educational attainment had higher risks for cancer death compared with race-specific counterparts with college education and low allostatic load. After adjustments for age, poverty-to-income ratio, smoking status, history of cancer, and ever congestive heart failure and heart attack, the associations were attenuated, but all men (HR, 1.69; 95% CI, 1.15-2.47) and White men (HR, 1.82; 95% CI, 1.16-2.85) still had greater than 50% increased risk of cancer death compared with men with college education and low allostatic load.

**Conclusions and Relevance:**

This study highlights the detrimental association of not attaining a high school degree, combined with high allostatic load as a marker of chronic stress, with cancer mortality. Efforts to promote educational attainment and address the underlying social determinants of health are imperative in reducing cancer disparities in this population.

## Key Points

**Question:**

Is allostatic load, a measure of chronic physiologic stress, associated with the risk of cancer mortality among men, and does educational attainment modify this association?

**Findings:**

In this cohort study of 20 529 men in the National Health and Nutrition Examination Survey from 1988 to 2910, those with high allostatic load and lower educational attainment had a greater than 4-fold higher risk of cancer mortality compared with those with low allostatic load and college educational attainment.

**Meaning:**

These findings suggest that efforts to promote educational attainment and address the underlying social determinants of health are imperative in reducing cancer disparities in this population.

## Teaser

This cohort study examines the association between allostatic load and cancer mortality risk in US men and whether educational attainment modifies the association.

## Introduction

In the US, the cancer incidence rate among male individuals is 497 cases per 100 000 population, with the highest incidence rate being among non-Hispanic Black men (544.0 cases per 100 000 population).^1^ Although lung cancer was the leading cause of male cancer deaths, incidence rates for prostate, pancreas, and kidney cancer among men increased between 2014 and 2018. Cancer death rates are also highest among non-Hispanic Black men (178.6 deaths per 100 000 population) compared with White (157.2 deaths per 100 000 population) and Hispanic (109.7 deaths per 100 000 population) men.

Social determinants of health, such as income, occupation, and education, can influence cancer disparities in men. Higher educational attainment can boost self-reported health in Black men, but the increase is smaller compared with White men.^2^ Men with limited access to education and income are more likely to have high allostatic load (AL), a measure of cumulative physiologic stress on the body over time. Chronic stress triggers prolonged neuroendocrine responses, resulting in elevated proinflammatory cytokines, catecholamines, and other proteins. This overactivation can lead to physiologic dysregulation and can promote cancer development.^3,4^ Moore et al^5^ found that high AL was associated with an increased risk of overall cancer death.

Non-Hispanic Black (hereafter, Black) men contend with a unique set of challenges and lived experiences that intricately intertwine with their health outcomes. Black men often navigate a landscape where socioeconomic disparities, limited access to quality education, and heightened exposure to chronic stressors converge owing to a pervasive and historical impact of systemic racism. Multiple studies have demonstrated how Black men consistently experience some of the highest levels of AL,^6,7,8^ which is associated with multiple negative health outcomes, including increased incidence of cancer, cardiovascular disease mortality, depression, and all-cause mortality.^9,10,11,12^ Cancer morbidity and mortality rates among Black men are of major concern, because they experience more aggressive disease at presentation and have higher rates of death from cancer compared with other demographic groups.^13,14,15^ This disparity highlights the urgent need for targeted interventions and equitable access to health care resources to address the underlying factors contributing to this disproportionate burden.

Current data are uncertain about the association between AL and education in Black men. Compared with their non-Hispanic White (hereafter, White) counterparts, Richardson et al^2^ found that for Black men, a college degree was deleterious and resulted in higher AL because, by the time they become young adults, their cumulative physiologic stress level is already higher than that of their White counterparts. However, focusing specifically on Black men, Rogers et al^16^ and Van Dyke et al^17^ found that those with college degrees had a lower prevalence of high AL than Black men with the lowest levels of education. The association between education and AL is not straightforward; Assari et al^18^ found that market preferences and behavioral factors also play a large role in Black men’s perception of their health. This only highlights the complicated association between AL and education within Black men and the need for further study.

Against this backdrop, the role of education emerges as a potential moderator, a factor that may either exacerbate or mitigate the impact of AL on health outcomes for men. Studies that have focused specifically on prostate cancer in men have suggested that there is an association between increased years of education and improved health.^19^ Therefore, it is important to better elucidate the modulating role of educational levels in men. The purpose of this study is to examine the association between education and AL and subsequent risk of cancer death, and whether these outcomes were differential by race and ethnicity.

## Methods

A retrospective cohort analysis was performed using data from the National Health and Nutrition Examination Survey (NHANES) linked with data from the National Center for Health Statistics (NCHS) 2019 National Death Index (NDI) file. The institutional review boards of the University of Kentucky and Augusta University considered this study exempt from review and the need for informed consent because this study uses secondary, publicly available, and deidentified data that do not meet the criteria for human participants research. This study follows the Strengthening the Reporting of Observational Studies in Epidemiology (STROBE) reporting guidelines.

The NHANES program oversamples those aged 60 years and older, Hispanic, and non-Hispanic Black individuals, and weighted analyses generate generalizable estimates.^20^ NHANES survey data from the years 1988 through 2010 linked with NDI data (follow-up data through December 31, 2019) containing questions related to sociodemographics, clinical measurements, and general health, were used for the project sample. Participants in NHANES self-identified their race and ethnicity, with categories of Black, Hispanic, White, and other including multiracial (NHANES does not further define the category of other).

NHANES participants with biomarker data and within a fasting subsample were eligible for the study (95 359 participants). Patients were excluded if they reported current pregnancy or were younger than 18 years (42 791 participants), were missing AL biomarkers, or were not linked via NDI (11 360 participants). Of participants over a 22-year study period, a final sample of NHANES male participants, assumed to be cisgender, aged 18 and older was included in the current analysis. Analyses to account for appropriate estimations of covariance-variance structures using specific strata, cluster, and weighting procedures as specified by NHANES methods were conducted. Mortality status or vital status for participants was determined through the NHANES-NDI linked file.

### Educational Attainment as an Effect Modifier

This study mirrored methods of the investigative team’s prior work.^21^ Educational attainment was analyzed as a modifier of the association between AL and cancer mortality, based on the NHANES question about the highest level of education completed. Education was categorized into 4 levels: (1) less than a high school (HS) education, (2) HS graduate or General Educational Development test, (3) some college, and (4) college graduate or higher.^21^ Specific degree types could not be distinguished within the NHANES data. In sensitivity analysis, education was also examined as a dichotomous variable, with high education (some college or higher) and low education (HS or less).

### AL as a Primary Independent Variable

AL has been defined using various methods, although most incorporate biomarker measures from 3 distinct categories, including physiologic functioning, which incorporates cardiovascular, metabolic, and immune systems.^22^ Although there is no consensus definition, we defined AL using the Geronimus et al^7^ and Moore et al^5^ taxonomies. AL components included body mass index, diastolic blood pressure, glycohemoglobin, systolic blood pressure, total cholesterol, serum triglycerides, serum albumin, serum creatinine, and C-reactive protein.

To determine the high-risk thresholds for each AL component, the sex reported at survey-specific distributions of each component among the entire study sample was examined with complete biomarker data (41 208 participants, inclusive of women and men from other racial groups). High-risk thresholds were determined by being either above the 75th percentile for body mass index, C-reactive protein, diastolic blood pressure, glycated hemoglobin, systolic blood pressure, total cholesterol, serum triglycerides, and serum creatinine,^23,24^ or below the 25th percentile for serum albumin. Therefore, each NHANES participant was scored as either 1 (high-risk) or 0 (low-risk) according to sex at baseline survey-specific cutoffs for each component. Total AL score was calculated by summing the individual components, ranging from 0 to 9. Participants were further categorized with AL scores greater or equal to 3 as having high AL.^22,25^

### Intersection of AL and Educational Attainment

After categorizing NHANES participants according to the distribution of AL components and their self-reported educational attainment, we created a variable examining the intersection of AL and educational attainment. This variable was categorized into 8 levels: (1) college graduate or more living with low AL, (2) college graduate or more living with high AL, (3) some college with low AL, (4) some college with high AL, (5) HS diploma or equivalent with low AL, (6) HS diploma or equivalent with high AL, (7) less than HS with low AL, and (8) less than HS with high AL.

### Primary Outcome of Interest: Time to Cancer Death

The primary outcome of interest was the time to cancer death, defined by malignant neoplasm–related deaths (*International Statistical Classification of Diseases and Related Health Problems, Tenth Revision *codes 019-043). Mortality data, available through December 31, 2019, came from NDI-NHANES publicly available linkages. Mortality for NHANES participants was determined by matching survey records to the NDI, although additional redundant sources are also incorporated, including the Social Security Administration, the Centers for Medicare & Medicaid Services, the NCHS’s follow-up surveys (eg, the NHANES Epidemiologic Follow-up Study), death certificates, and additional data collection. In competing risk analysis, all other causes of death were treated as competing events.

### Other Variables of Interest

Other covariates were included as potential confounders or for their possible associations with education, cumulative stress, and cancer outcomes, on the basis of prior research. These variables included NHANES survey periods (1988-1991 through 2009-2010), family poverty-to-income ratio (PIR), current smoking status, and self-reported history of cancer, congestive heart failure, and or heart attack. PIR was calculated as the ratio of total family income to poverty threshold, with values above 1 indicating income above the poverty line and those below 1 indicating income below the poverty line.^21^ Current smokers were defined as participants who had smoked at least 100 cigarettes in their lifetime and were smoking during the survey. Diagnoses of cancer, congestive heart failure, or heart attack were self-reported from NHANES questionnaires.

### Statistical Analysis

Primary analyses used NHANES sampling strata, clusters, and weights, following guidelines from the NHANES methods handbook.^26,27,28^ Since biomarkers are measured in random subsamples, subsample weights were adjusted for selection probability and nonresponse bias. Data from NHANES 1988 to 1994 and 1999 to 2010 were combined, adjusting sample weights to cover 18 years. The mobile examination center included physical measurements, such as blood pressure, a dental examination, and the collection of blood and urine specimens for laboratory testing. Following analytic guidelines by the NCHS,^29,30,31^ the NHANES mobile examination center sample weights were used for NHANES III (years 1988-1994) and NHANES 1999 to 2010, and were constructed an adjusted weight by modifying the weights to have a common denominator of 18; that is, (1) the 6-year weight for 1988 to 1994 was multiplied by one-third, (2) the 4-year weight for 1999 to 2002 was multiplied by one-fourth and a half, and (3) the 2-year cycle weights for each subsequent NHANES (years 2003-2010) were multiplied by one-ninth. Assumptions include no differences in estimates over time, estimates are the average over the period, and NHANES III (1988-1994) recruited noninstitutionalized, US individuals aged 2 months and older, whereas the continuous survey (1999 and later) recruited all ages.

Categorical variables were presented as weighted row percentages, and continuous variables were presented as mean and associated SE estimates using appropriate survey-weighted procedures.^32^ Mean survival times were estimated using the product-limit method of the Kaplan-Meier survival estimator. Proportionality assumption was assessed as the primary variable of interest (education attainment by AL status) by examining the proportion of 1000 simulations that contained a maximum cumulative martingale residual larger than the observed maximum cumulative residuals using the SAS procedure supremum test. None of the exposure levels had *P *values that were statistically significant (*P* < .05); therefore, no residuals were larger than expected and the proportional hazards assumptions were not rejected.

Relative rates of cancer death by groups of educational attainment and AL were estimated by fitting survey-weighted Cox proportional hazards models with time to cancer death as the end point. Individuals were censored at the time of their event, death, or end of follow-up (December 31, 2019). Models were sequentially adjusted (1) for age and then further with (2) age, PIR, smoking status, ever cancer history, ever congestive heart failure, and ever heart attack.

We conducted a competing risk analysis as a sensitivity analysis to account for the possibility that other causes of death could compete with cancer mortality, influencing the observed associations. For the competing risk analysis, we used the Fine and Gray subdistribution hazard model, which accounts for competing events by modeling the cumulative incidence of the event of interest, while considering competing events.^33^ This model allows the risk of cancer mortality in the presence of other potential causes of death to be estimated, which provides a more realistic and nuanced understanding of the association between AL and cancer mortality.

Multiplicative interactions of AL and education were tested by adding an interaction term to the model, with a 2-tailed *P* ≤ .05, determined by the Rao-Scott χ^2^ test, considered statistically significant. Time to cancer death event survival analyses, stratified by AL status (high vs low), were also performed. Estimates were presented as hazard ratios (HRs) with 95% CIs from survey-weighted Cox models. We analyzed (1) the joint association of education and AL with cancer death risk and (2) how education moderated the AL-cancer death risk association. All statistical analyses were performed using SAS statistical software version 9.4 (SAS Institute). Data were analyzed from June to October 2024.

## Results

The final sample included 20 529 men (Figure), representing 84 363 491 US men from 1988 to 2010: 4418 Black men (21.5%), 5762 Hispanic men (28.1%), 9556 White men (46.5%), and 793 men (3.9%) of other races, including multiracial. The mean (SE) age was 41.00 (0.22) years. In total, 2065 participants were college graduates with low AL, 1350 were college graduates with high AL, 2338 had some college and low AL, 1795 had some college and high AL, 3129 had a HS diploma or equivalent and low AL, 2385 had a HS diploma or equivalent and high AL, 3698 had less than HS and low AL, and 3699 had less than HS and high AL. White men were more likely to have greater than college with high AL (9.39%) compared with Black (5.4%) and Hispanic (2.90%) men. In the referent group (greater than college with low AL), most were White (19.06%), followed by Hispanic (5.78%) and Black (6.66%) men. Hispanic (17.31%) and Black (16.47%) men were more likely to have less than HS education with high AL, whereas low AL in this group was also more common in in Hispanic (33.22%) and Black (15.01%) men. Men in the HS or General Educational Development with low AL group had the highest smoking rates (20.80%), and those with less than HS and high AL had the highest congestive heart failure (28.49%) and heart attack history (24.72%) rates (Table 1). Mean AL sum scores tended to decrease with educational attainment (eTable 1 in Supplement 1) among all men and White men; however, among Black men and Hispanic men, the mean AL scores followed a U-shaped relationship with similar AL scores for those with less than HS and college education.

**Figure.**
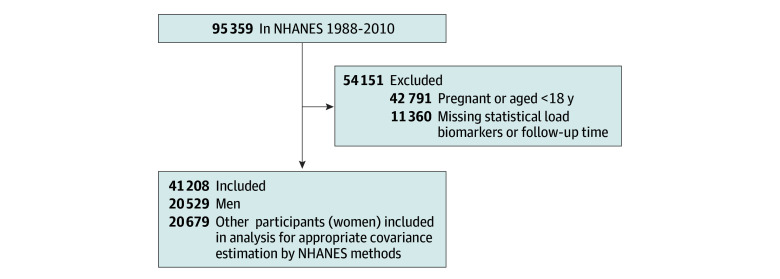
Flowchart of Exclusion Criteria and Final Study Population of National Health and Nutrition Examination Survey (NHANES) Participants

**Table 1.** Sample Characteristics by Allostatic Load and Educational Attainment Among Men Surveyed by National Health and Nutrition Examination Survey, 1988-2010^a^

Characteristic	Participants, unweighted % (weighted %)								*P* value^c^
	High allostatic load^b^				Low allostatic load				
	Less than HS	HS or GED	Some college	College graduate	Less than HS	HS or GED	Some college	College graduate	
Unweighted sample size	3699	2385	1795	1350	3698	3129	2338	2065	NA
Weighted sample size	8 388 836	9 331 205	7 901 455	7 013 095	10 044 443	14 330 669	13 275 635	13 936 664	NA
Allostatic load total score, mean (SE)^d^	4.11 (0.28)	4.02 (0.03)	3.99 (0.03)	3.81 (0.03)	1.07 (0.02)	1.05 (0.02)	1.05 (0.02)	1.02 (0.02)	<.001
Age, mean (SE), y	54.62 (0.48)	50.08 (0.43)	49.54 (0.46)	52.68 (0.57)	38.76 (0.47)	36.95 (0.38)	38.20 (0.42)	43.28 (0.44)	<.001
Race and ethnicity									
Non-Hispanic Black	16.47 (0.69)	14.46 (0.63)	11.80 (0.63)	5.64 (0.45)	15.01 (0.53)	16.09 (0.73)	13.54 (0.62)	6.66 (0.53)	<.001
Non-Hispanic White	7.72 (0.44)	11.53 (0.44)	9.79 (0.35)	9.39 (0.44)	8.15 (0.54)	17.61 (0.59)	16.62 (0.46)	19.06 (0.96)	
Hispanic	17.31 (0.62)	6.96 (0.50)	6.05 (0.53)	2.90 (0.34)	8.15 (0.50)	15.31 (0.77)	12.27 (0.77)	5.78 (0.51)	
Other including multiracial	13.11 (2.06)	7.52 (1.19)	6.42 (1.11)	9.36 (1.15)	13.66 (1.49)	13.92 (1.66)	14.86 (1.49)	29.93 (2.07)	
Age group, y									
18-29	20.26 (0.89)	4.92 (0.43)	3.74 (0.46)	1.36 (0.27)	3.73 (0.36)	28.46 (1.08)	24.40 (0.88)	12.95 (0.91)	<.001
30-39	11.87 (0.77)	9.08 (0.60)	7.28 (0.56)	6.06 (0.70)	6.31 (0.52)	19.90 (1.09)	18.36 (1.17)	20.94 (1.15)	
40-49	8.43 (0.58)	12.56 (0.84)	12.31 (0.77)	10.39 (0.77)	8.22 (0.66)	14.19 (0.78)	13.58 (0.75)	20.18 (1.43)	
50-59	7.34 (0.57)	14.52 (0.88)	13.45 (0.99)	12.52 (0.90)	12.02(0.80)	10.17 (0.74)	13.31 (0.85)	16.50 (1.12)	
60-69	8.18 (0.68)	15.74 (0.99)	12.38 (0.79)	14.31 (0.94)	18.33 1.08)	9.61 (0.78)	7.26 (0.68)	14.13 (1.16)	
≥70	10.90 (0.78)	16.29 (0.83)	11.14 (0.67)	12.05 (0.76)	24.42(1.06)	8.00 (0.55)	6.84 (0.54)	10.06 (0.87)	
Time period^e^									
1988-1991	943 (14.67)	504 (12.67)	260 (6.86)	220 (7.83)	718 (12.14)	531 (18.21)	327 (13.53)	261 (13.72)	<.001
1991-1994	955 (14.63)	569 (16.21)	300 (9.88)	255 (11.40)	515 (10.21)	410 (14.48)	246 (10.14)	207 (12.83)	
1999-2002	525 (7.59)	328 (7.99)	281 (7.74)	237 (7.18)	900 (13.88)	695 (18.65)	535 (18.14)	495 (18.42)	
2003-2006	516 (7.24)	393 (9.94)	393 (10.68)	258 (8.10)	736 (11.29)	706 (17.72)	564 (18.14)	453 (16.84)	
2007-2010	760 (8.55)	591 (10.46)	561 (10.79)	380 (7.80)	829 (11.69)	787 (15.77)	666 (16.21)	649 (18.62)	
Family poverty-to-income ratio, mean (SE)	2.08 (0.04)	2.99 (0.06)	3.42 (0.07)	4.28 (0.07)	1.97 (0.05)	2.91 (0.05)	3.26 (0.06)	4.13 (0.05)	<.001
Current smoker status	12.54 (0.76)	13.64 (0.67)	8.70 (0.51)	3.80 (0.32)	17.50 (0.70)	20.80 (0.72)	15.36 (0.77)	7.45 (0.66)	<.001
Any cancer history^f^	13.59 (1.08)	13.68 (1.22)	13.19 (1.11)	15.41 (1.78)	8.20 (0.89)	10.59 (1.36)	9.81 (1.09)	15.41 (0.75)	<.001
Obesity-related cancer	15.66 (3.25)	11.13 (2.94)	25.27 (5.60)	18.87 (5.24)	7.55 (2.37)	8.06 (2.29)	6.00 (2.22)	7.45 (2.72)	<.001
Congestive heart failure	28.49 (2.11)	19.47 (2.18)	12.87 (1.70)	11.19 (1.97)	8.76 (1.36)	6.02 (1.35)	7.81 (1.50)	5.22 (1.24)	<.001
History of heart attack	24.72 (1.43)	17.99 (1.60)	13.52 (1.28)	10.57 (1.05)	9.37 (1.13)	8.50 (1.16)	7.24 (0.88)	7.76 (1.16)	<.001

Abbreviations: GED, General Educational Development; HS, high school; NA, not applicable.

^a^ Data are from the National Health Examination Survey (1988-2010 with follow-up through December 31, 2019).

^b^ High allostatic load is defined as total allostatic load score greater than or equal to 3 (data are presented as column percentages and SEs).

^c^ *P* values were determined using Rao-Scott χ^2^ tests.

^d^ Estimated using sampling weights from National Health and Nutrition Examination Survey.

^e^ Presented as unweighted row sample size (weighted percentage).

^f^ Defined as self-reported response to ever being diagnosed by a doctor or health professional of any cancer or malignant neoplasm.

There were 4638 all-cause deaths in the cohort. Among all men, those with high AL and less than HS educational attainment had a greater than 4-fold increased risk of cancer mortality (unadjusted HR, 4.71; 95% CI, 3.36-6.60) (Table 2) compared with those with low AL and a college degree or higher. After adjusting for age, the same group had a 2-fold increased risk of cancer (age-adjusted HR, 2.35; 95% CI, 1.66-3.32). Specifically among White men, participants with less than HS education with high AL had a more than 5-fold increased risk of cancer death (unadjusted HR, 5.77; 95% CI, 4.06-8.20) compared with White men with college education and low AL. Furthermore, after adjusting for age (HR, 2.55; 95% CI, 1.72-3.79) and also for PIR, smoking status, cancer, time period, ever congestive heart failure, and ever heart attack (HR, 1.82; 95% CI, 1.16-2.85), White men with less than HS education with high AL were still at increased risk of dying from cancer compared with White men with college education and low AL. Similarly, among Black men, participants with less than HS education and high AL (unadjusted HR, 4.19; 95% CI, 2.09-8.40) were at greater than 4-fold increased risk for cancer death compared with those Black men with college education and low AL. When adjusted for age (HR, 1.58; 95% CI, 0.81-3.10), and further adjusted for other confounders (HR, 1.09; 95% CI, 0.54-2.22) the HRs were no longer significant.

**Table 2.** Risk of Cancer Death Associated With Educational Attainment and AL Status and Stratified by Race^a^

Racial and ethnic group, educational attainment, and AL status	No. at risk, unweighted^c^	No. of cancer deaths (weighted %)^d^	Survival, mean (SE), mo^e^	HR (95% CI)^b^		
				Unadjusted	Age adjusted	Fully adjusted^f^
All men						
College graduate						
Low AL	2065	94 (3.23)	281.9 (0.81)	1 [Reference]	1 [Reference]	1 [Reference]
High AL	1350	101 (5.51)	264.6 (1.28)	1.74 (1.23-2.44)	0.94 (0.67-1.32)	0.86 (0.61-1.23)
Some college						
Low AL	2338	92 (2.69)	331.7 (1.07)	0.84 (0.58-1.21)	1.21 (0.84-1.73)	0.98 (0.68-1.43)
High AL	1795	152 (6.70)	292.4 (1.54)	2.40 (1.70-3.37)	1.65 (1.14-2.38)	1.31 (0.88-1.94)
HS or GED						
Low AL	3129	118 (3.21)	338.3 (0.78)	0.95 (0.64-1.42)	1.32 (0.91-1.92)	1.02 (0.70-1.49)
High AL	2385	251 (9.60)	290.4 (1.49)	3.20 (2.29-4.49)	2.20 (1.59-3.06)	1.63 (1.17-2.27)
Less than HS						
Low AL	3698	192 (4.94)	338.2 (0.95)	1.55 (1.13-2.12)	1.70 (1.23-2.35)	1.17 (0.83-1.66)
High AL	3699	491 (12.70)	307.1 (1.74)	4.71 (3.36-6.60)	2.35 (1.66-3.32)	1.69 (1.15-2.47)
*P* value for interaction between education and AL	NA	NA	NA	.01	.10	.04
White men						
College graduate						
Low AL	1468	71 (3.4)	282.3 (0.9)	1 [Reference]	1 [Reference]	1 [Reference]
High AL	901	80 (6.1)	263.4 (1.7)	1.81 (1.25-2.60)	0.99 (0.69-1.41)	0.90 (0.62-1.29)
Some college						
Low AL	1288	59 (2.8)	280.3 (1.2)	0.83 (0.54-1.29)	1.17 (0.77-1.77)	0.94 (0.61-1.44)
High AL	956	98 (7.3)	287.1 (2.5)	2.51 (1.76-3.58)	1.70 (1.17-2.48)	1.32 (0.88-1.98)
HS or GED						
Low AL	1534	77 (3.5)	276.8 (0.9)	0.99 (0.64-1.54)	1.31 (0.87-1.97)	0.99 (0.65-1.50)
High AL	1232	162 (10.7)	272.6 (2.3)	3.48 (2.45-4.94)	2.32 (1.64-3.28)	1.72 (1.22-2.44)
Less than HS						
Low AL	933	79 (7.0)	294.9 (2.3)	2.08 (1.42-3.05)	1.83 (1.24-2.72)	1.20 (0.79-1.82)
High AL	1224	193 (15.5)	265.3 (3.2)	5.77 (4.06-8.20)	2.55 (1.72-3.79)	1.82 (1.16-2.85)
*P* value for interaction between education and AL	NA	NA	NA	.04	.12	.04
Black men						
College graduate						
Low AL	229	11 (3.8)	189.5 (1.8)	1 [Reference]	1 [Reference]	1 [Reference]
High AL	232	16 (5.2)	224.9 (2.4)	1.44 (0.62-3.38)	0.87 (0.38-1.99)	0.93 (0.42-2.08)
Some college						
Low AL	464	24 (3.9)	272.4 (1.7)	1.04 (0.52-2.09)	1.48 (0.74-2.94)	1.34 (0.67-2.70)
High AL	485	37 (6.0)	246.2 (2.1)	1.69 (0.75-3.82)	1.01 (0.45-2.27)	0.91 (0.40-2.09)
HS or GED						
Low AL	701	31 (3.7)	286.9 (1.3)	0.92 (0.41-2.07)	1.28 (0.58-2.80)	1.02 (0.45-2.33)
High AL	652	57 (6.4)	290.3 (2.3)	1.62 (0.79-3.33)	1.13 (0.54-2.33)	0.89 (0.42-1.88)
Less than HS						
Low AL	690	37 (4.7)	246.6 (1.4)	1.24 (0.57-2.71)	1.42 (0.68-2.96)	0.93 (0.42-2.06)
High AL	943	155 (13.3)	294.2 (3.5)	4.19 (2.09-8.40)	1.58 (0.81-3.10)	1.09 (0.54-2.22)
*P* value for interaction between education and AL	NA	NA	NA	.11	.60	.43
Hispanic men						
College graduate						
Low AL	228	10 (2.2)	200.1 (1.5)	1 [Reference]	1 [Reference]	1 [Reference]
High AL	141	4 (1.2)	202.5 (2.2)	0.71 (0.20-2.56)	0.39 (0.11-1.42)	0.36 (0.10-1.37)
Some college						
Low AL	476	9 (2.2)	334.6 (2.0)	1.00 (0.36-2.65)	1.59 (0.54-4.67)	1.17 (0.38-3.56)
High AL	302	14 (2.1)	292.3 (2.4)	1.01 (0.39-2.65)	0.84 (0.30-2.33)	0.73 (0.27-1.99)
HS or GED						
Low AL	766	8 (0.6)	344.6 (0.3)	0.25 (0.08-0.82)	0.51 (0.15-1.69)	0.43 (0.14-1.31)
High AL	434	27 (3.2)	301.4 (2.4)	1.44 (0.59-3.48)	1.07 (0.44-2.61)	0.91 (0.38-2.17)
Less than HS						
Low AL	1963	72 (2.0)	342.6 (1.0)	0.84 (0.36-1.95)	1.11 (0.44-2.79)	0.82 (0.30-2.21)
High AL	1426	137 (6.1)	320.4 (2.2)	2.91 (1.32-9.17)	1.42 (0.61-3.31)	1.07 (0.43-2.65)
*P* value for interaction between education and AL	NA	NA	NA	.01	<.001	.01
Other (including multiracial) men						
College graduate						
Low AL	140	2 (1.4)	39.0 (NA)^g^	1 [Reference]	1 [Reference]	1 [Reference]
High AL	76	1 (0.8)	122.0 (NA)^g^	0.59 (0.05-7.77)	0.28 (0.02 3.80)	0.33 (0.03-4.00)
Some college						
Low AL	110	0 (0.0)	NA^g^	NA^g^	NA^g^	NA^g^
High AL	52	3 (5.0)	65.8 (1.6)	3.78 (0.46-30.91)	2.40 (0.29-20.13)	2.49 (0.33-18.54)
HS or GED						
Low AL	128	2 (2.9)	232.8 (2.2)	1.62 (0.19-13.58)	1.86 (0.26-13.19)	2.22 (0.38-12.88)
High AL	67	5 (10.1)	291.6 (8.8)	6.70 (0.89-50.33)	3.49 (0.46-26.75)	1.30 (0.24-7.06)
Less than HS						
Low AL	112	4 (3.5)	188.4 (1.4)	1.94 (0.27-13.79)	1.77 (0.25-12.66)	1.70 (0.26-11.24)
High AL	106	6 (7.2)	231.8 (3.3)	4.04 (0.69-23.68)	2.25 (0.33-15.40)	1.81 (0.28-1.89)
*P* value for interaction between education and AL	NA	NA	NA	.01	.01	.01

Abbreviations: AL, allostatic load; GED, General Educational Development; HR, hazard ratio; HS, high school; NA, not applicable.

^a^ Data are from the National Health Examination Survey (1988-2010 with follow-up through December 31, 2019).

^b^ HRs were determined using survey-weighted Cox proportional hazards model.

^c^ The sample included 20 529 NHANES men participants with 1501 cancer-related deaths.

^d^ Percentages are weighted. Cox proportional hazard models are estimated using NHANES survey weighting. The weighted population included 84 363 491 men and 4 657 724 cancer-related deaths.

^e^ Mean survival months are unweighted.

^f^ Fully adjusted for age, family poverty-to-income ratio, current smoker status, cancer, time period, congestive heart failure, and heart attack.

^g^ Indicates null result owing to small number in analysis.

In Table 3, we present models stratified by race and ethnicity and education When stratified among Hispanic men with HS education, those men with high AL had a greater than 5-fold increased risk of cancer mortality, the highest risk among all racial and ethnic groups (unadjusted HR, 5.77; 95% CI, 2.05-16.19). Similarly, among Black men with less than HS education, those with high AL had a 3-fold increased risk of cancer mortality (unadjusted HR, 3.38; 95% CI, 2.16-5.26) compared with Black men with low AL. Among White men, those with less than HS education and high AL (fully adjusted HR, 1.69; 95% CI, 1.15-2.47) and with HS education and high AL (fully adjusted HR, 1.65; 95% CI, 1.14-2.37) had higher risk of cancer mortality compared with their counterparts with low AL.

**Table 3.** AL Status and Association With Risk of Cancer Death, Stratified by Educational Attainment and Race

Racial and ethnic group, educational attainment, and AL status	No. of cancer deaths (weighted %)^b^^,^^c^	HR (95% CI)^a^		
		Unadjusted	Age adjusted	Fully adjusted^d^
Black				
College graduate				
Low AL	11 (3.8)	1 [Reference]	1 [Reference]	1 [Reference]
High AL	16 (5.2)	1.46 (0.63-3.42)	0.81 (0.35-1.92)	1.06 (0.42-2.66)
Some college				
Low AL	24 (3.9)	1 [Reference]	1 [Reference]	1 [Reference]
High AL	37 (6.0)	1.62 (0.90-2.92)	0.69 (0.39-1.25)	0.67 (0.38-1.19)
HS or GED				
Low AL	31 (3.7)	1 [Reference]	1 [Reference]	1 [Reference]
High AL	57 (6.4)	1.75 (1.12-2.75)	0.83 (0.55-1.27)	0.76 (0.47-1.22)
Less than HS				
Low AL	37 (4.7)	1 [Reference]	1 [Reference]	1 [Reference]
High AL	155 (13.3)	3.38 (2.16-5.26)	1.15 (0.75-1.75)	1.25 (0.80-1.96)
White				
College graduate				
Low AL	71 (3.4)	1 [Reference]	1 [Reference]	1 [Reference]
High AL	80 (6.1)	1.82 (1.27-2.61)	0.92 (0.65-1.31)	0.87 (0.60-1.25)
Some college				
Low AL	59 (2.8)	1 [Reference]	1 [Reference]	1 [Reference]
High AL	98 (7.3)	2.99 (2.05)	1.35 (0.93-1.95)	1.39 (0.97-2.01)
HS or GED				
Low AL	77 (3.5)	1 [Reference]	1 [Reference]	1 [Reference]
High AL	162 (10.7)	3.55 (2.40-5.23)	1.74 (1.19-2.53)	1.65 (1.14-2.37)
Less than HS				
Low AL	79 (7.0)	1 [Reference]	1 [Reference]	1 [Reference]
High AL	193 (15.5)	2.73 (1.91-3.91)	1.53 (1.08-2.15)	1.69 (1.17-2.44)
Hispanic				
College graduate				
Low AL	10 (2.2)	1 [Reference]	1 [Reference]	1 [Reference]
High AL	4 (1.2)	0.70 (0.20-2.53)	0.33 (0.10-1.21)	0.25 (0.06-1.11)
Some college				
Low AL	9 (2.2)	1 [Reference]	1 [Reference]	1 [Reference]
High AL	14 (2.1)	1.03 (0.41-2.57)	0.50 (0.20-1.27)	0.76 (0.30-1.92)
HS or GED				
Low AL	8 (0.6)	1 [Reference]	1 [Reference]	1 [Reference]
High AL	27 (3.2)	5.77 (2.05-16.19)	2.85 (1.34-7.12)	2.50 (0.96-6.52)
Less than HS				
Low AL	72 (2.0)	1 [Reference]	1 [Reference]	1 [Reference]
High AL	137 (6.1)	3.49 (2.06-5.91)	1.34 (0.73-2.44)	1.36 (0.70-2.66)
Other race or multiracial				
College graduate				
Low AL	2 (1.4)	1 [Reference]	1 [Reference]	1 [Reference]
High AL	1 (0.8)	0.56 (0.04-7.10)	0.22 (0.01-3.62)	0.23 (NA)^e^
Some college				
Low AL	0 (0.0)	1 [Reference]	1 [Reference]	1 [Reference]
High AL	3 (5.0)	NA^e^	NA^e^	NA^e^
HS or GED				
Low AL	2 (2.9)	1 [Reference]	1 [Reference]	1 [Reference]
High AL	5 (10.1)	4.15 (0.83-20.84)	2.26 (0.77-6.65)	0.92 (NA)^e^
Less than HS				
Low AL	4 (3.5)	1 [Reference]	1 [Reference]	1 [Reference]
High AL	6 (7.2)	2.13 (0.47-9.65)	1.39 (0.26-7.44)	1.10 (0.26-4.71)

Abbreviations: AL, allostatic load; HR, hazard ratio; NA, not applicable.

^a^ HRs are estimated using survey-weighted Cox proportional hazards model.

^b^ The sample included 20 529 NHANES men participants with 1501 cancer-related deaths.

^c^ Percentages are weighted. The weighted population includes 84 363 491 men and 4 657 724 cancer-related deaths.

^d^ Fully adjusted for age, family poverty to income ratio, current smoker status, cancer, time-period, congestive heart failure, and heart attack.

^e^ Indicates null result due to small number in analysis.

A competing risk analysis using the Fine-Gray proportional hazard model was conducted in unadjusted, age-adjusted, and fully adjusted models (eTable 2 in Supplement 1) showing similar effects across all models. Men with high AL and less than HS education had a significantly higher risk of cancer death compared with those with higher education levels and low AL, although this was attenuated in the age-adjusted and fully adjusted models. When education was dichotomized (low, education defined as HS education and less than HS education; and high, education defined as some college and college graduates) in eTables 3 and 4 in Supplement 1, men with low education and high AL had 65% increased risk of cancer death (fully adjusted HR, 1.65; 95% CI, 1.31-2.08) and 79% increased risk of cancer death among White men (fully adjusted HR, 1.79; 95% CI, 1.39-2.30) compared with high education, low AL counterparts (eTable 3 in Supplement 1). In most race-specific and education-specific models, associations were attenuated in fully adjusted models; however, among White men, those with low education and high AL still had 67% higher risk (fully adjusted HR, 1.67; 95% CI, 1.26-2.20) (eTable 4 in Supplement 1). Results from an unweighted cohort (treated as simple random sample) in eTable 5 in Supplement 1 mirrored those of NHANES weighted analysis, but effect measures were less pronounced.

## Discussion

This cohort study explored the associations between AL and educational attainment in men, as well as their association with cancer mortality. The findings indicate that men with lower educational attainment and high AL face up to a 4-fold increased risk of cancer mortality and nearly 70% increased risk of cancer mortality when accounting possible confounders. In addition, when adjusted for age, chronic physiological stress is greatly associated with higher cancer mortality in all men without a HS degree. These findings suggest the importance of finishing HS to reduce the likelihood of cancer mortality in the population of interest. Without a HS education, men may experience limited economic opportunities, leading to poverty, lack of health insurance, and reduced access to health care services in general.

The current study aligns with previous research that identifies education level as a social determinant of health, showing a correlation between educational attainment and lower AL.^34^ This is consistent with the findings of Rogers et al,^16^ who observed that Black male college graduates tend to have lower AL compared with those with less than a HS education. Similarly, this study revealed that obtaining a HS degree was associated with reduced AL and a decrease in cancer mortality, before adjusting for age. These findings emphasize the potential health advantages that may accompany the completion of a HS education. By expanding access to education among men, we may contribute to improving health outcomes and reducing the burden of cancer in this population.

We found that cancer mortality inequities persisted for White men according to education and AL, but attenuated for Black men after adjusting for factors like age. Despite higher education, Black men face chronic stressors from systemic racism, such as discrimination, economic insecurity, and limited health care, that increase AL. Although education offers some socioeconomic advantages, it cannot fully protect against the health impact of structural racism. Black men face added stress, from microaggressions to overt racism, that may contribute to increased AL. Education helps reduce the association between AL and cancer mortality but it alone cannot counter the effects of systemic inequities. Expanding access to education and health resources for Black men could reduce stressors, promote cancer prevention, and delay cancer onset.

Gilbert et al^35^ found that, regardless of income, Black men are 50% less likely than White men to have seen a physician in the past year. Although higher education is typically linked to higher income, health literacy, and health care access, this may not apply to Black men. This could explain why cancer mortality rates do not vary much among Black men with higher education. Future research should focus on health promotion campaigns tailored to Black men, emphasizing education’s role in reducing cancer mortality and raising awareness about AL, cancer outcomes, and resources for education and health care.

### Limitations and Strengths

The limitations of this study must be noted. First, individuals with high AL were older than those with lower AL, and age is a key confounder in the associations among education, AL, and cancer death risk. Other limitations include potential measurement errors in the NHANES data that may have influenced the accuracy of the exposure and outcome variables, potentially leading to misclassification bias. Additionally, violations of the weighting assumption could affect the generalizability of the findings, as these weights account for survey design and nonresponse, but violations could introduce bias in the estimates.

Selection bias is also a concern owing to the exclusion of participants with missing exposure or outcome data, which could lead to a nonrepresentative sample and skew results. To address these limitations, efforts were made to conduct sensitivity analyses and imputation methods to minimize the impact of missing data and examine the robustness of the findings. The likely direction and magnitude of these biases were considered, and although some may attenuate effect estimates, others could potentially exaggerate them.

This study also possesses notable strengths. We used NHANES data from 1988 to 2010 because they provided complete AL variables, unlike newer datasets. Although recent data could offer updated insights, they lack the necessary variables for our analysis. A key strength of this study is its use of a nearly decade-long longitudinal dataset, unlike traditional datasets that cover a short period. However, AL and other relevant factors were assessed only once, limiting analysis of change over time. Despite this, the nationally representative sample allows for the generalizability of findings. This study also addresses the understudied associations among AL, education, and cancer mortality in men, an interaction that has long been understudied.

## Conclusions

This study found an increased risk of cancer mortality among men with lower educational attainment and high AL. Education, particularly the completion of HS, plays a crucial role in modifying the association between AL and cancer mortality. To address cancer disparities in the population with low educational achievement, it is essential to prioritize educational attainment and address the underlying social determinants of health. Therefore, it is vital to invest in policies and programs that improve educational access, especially for underserved, minoritized racial and ethnic groups. By empowering men with better access to resources that promote health and address chronic and acute life stressors, we can potentially reduce their vulnerability to cancer. When replicating the study, consideration should be given to changes in AL over time, access to health care, and other sociodemographic factors. Establishing this causal connection could guide targeted interventions and policies to effectively address disparities, reduce educational gaps, improve health care access, and decrease cancer mortality rates among men with lower educational attainment.

## Notes

Supplement 1. **eTable 1.** Mean allostatic load score by educational attainment, overall and by race/ethnicity among 20,529 men surveyed by NHANES years 1988–2010 **eTable 2.** Competing risks analysis to examine the association between educational attainment and allostatic load status with risk of cancer death, conducting using Fine & Gray Cox proportional hazards model **eTable 3.** Risk of cancer death associated with educational attainment (dichotomous) and allostatic load status low/high groups and stratified by race **eTable 4.** Association between allostatic load status and risk of cancer death, stratified by educational attainment (dichotomous) and race **eTable 5.** Unweighted analysis (treated as simple random sample) of risk of cancer death associated with educational attainment and allostatic load status and stratified by race

Supplement 2.
Data Sharing Statement
