# Supplementary material for: Allostatic Load, Educational Attainment, and Risk of Cancer Mortality Among US Men
Source: JAMA Netw Open. 2024 Dec 10;7(12):e2449855. doi: 10.1001/jamanetworkopen.2024.49855 (PMC11632542; doi:10.1001/jamanetworkopen.2024.49855)
Supplement: Supplement 1. — eTable 1. Mean allostatic load score by educational attainment, overall and by race/ethnicity among 20,529 men surveyed by NHANES years 1988–2010 eTable 2. Competing risks analysis to examine the association between educational attainment and allostatic load status with risk of cancer death, conducting using Fine & Gray Cox proportional hazards model eTable 3. Risk of cancer death associated with educational attainment (dichotomous) and allostatic load status low/high groups and stratified by race eTable 4. Association between allostatic load status and risk of cancer death, stratified by educational attainment (dichotomous) and race eTable 5. Unweighted analysis (treated as simple random sample) of risk of cancer death associated with educational attainment and allostatic load status and stratified by race [file jamanetwopen-e2449855-s001.pdf]

Supplemental Online Content

Li C, Howard SP, Rogers CR, et al. Allostatic load, educational attainment, and risk of cancer mortality among US men. *JAMA Netw Open*. 2024;7(12):e2449855. doi:10.1001/jamanetworkopen.2024.49855

eTable 1. Mean allostatic load score by educational attainment, overall and by race/ethnicity among 20,529 men surveyed by NHANES years 1988–2010

eTable 2. Competing risks analysis to examine the association between educational attainment and allostatic load status with risk of cancer death, conducting using Fine & Gray Cox proportional hazards model

eTable 3. Risk of cancer death associated with educational attainment (dichotomous) and allostatic load status low/high groups and stratified by race

eTable 4. Association between allostatic load status and risk of cancer death, stratified by educational attainment (dichotomous) and race

eTable 5. Unweighted analysis (treated as simple random sample) of risk of cancer death associated with educational attainment and allostatic load status and stratified by race

This supplemental material has been provided by the authors to give readers additional information about their work.

© 2024 Li C et al. *JAMA Network Open.*

**eTable 1: Mean allostatic load score by educational attainment, overall and by race/ethnicity among 20,529 men surveyed by NHANES years 1988 – 2010.**					
	**Educational Attainment Level**				
	**Less than HS**	**HS or GED**	**Some College**	**College Graduate**	**P value**
**Among All participants**					
Mean AL (SD)	2.63 (1.85)	2.36 (1.79)	2.39 (1.76)	2.21 (1.66)	<0.0001
**Among NH-White Men**					
Mean AL (SD)	2.90 (1.83)	2.40 (1.77)	2.31 (1.70)	2.14 (1.65)	<0.0001
**Among NH-Black Men**					
Mean AL (SD)	2.99 (1.90)	2.62 (1.80)	2.79 (1.81)	2.74 (1.72)	<0.0001
**Among Latino/Other Hispanic Men**					
Mean AL (SD)	2.40 (1.79)	2.01 (1.76)	2.23 (1.79)	2.12 (1.58)	<0.0001
**Among Other/Mixed Race Men**					
Mean AL (SD)	2.50 (1.80)	2.11 (1.75)	1.93 (1.74)	1.99 (1.54)	0.0034
P values determined weighted F-tests generated from Analysis of Variance (ANOVA) analysis.					

© 2024 Li C et al. *JAMA Network Open.*

eTable 2. Competing risks analysis to examine the association between educational attainment and allostatic load status with risk of cancer death[a], conducting using Fine & Gray Cox proportional hazards model[b]

Educational Attainment and Allostatic Load (AL) Status	Hazard Ratio[c] (95% CI)		
	Unadjusted	Age-Adjusted	Fully Adjusted[d]
**All Men**			
College graduate, Low AL	1.00 (Referent)	1.00 (Referent)	1.00 (Referent)
College graduate, High AL	1.57 (1.18–2.07)	0.99 (0.74–1.32)	0.88 (0.66 – 1.17)
Some college, Low AL	0.85 (0.64–1.13)	1.12 (0.84–1.49)	1.02 (0.76 – 1.36)
Some college, High AL	1.86 (1.44–2.40)	1.40 (1.08–1.81)	1.20 (0.92 – 1.56)
HS or GED, Low AL	0.78 (0.60–1.02)	1.12 (0.85–1.46)	0.97 (0.74 – 1.27)
HS or GED, High AL	2.13 (1.68–2.70)	1.60 (1.26–2.03)	1.29 (1.01 – 1.65)
Less than HS, Low AL	1.05 (0.82–1.35)	1.20 (0.94–1.54)	0.97 (0.75 – 1.27)
Less than HS, High AL	2.75 (2.21–3.43)	1.58 (1.26–1.98)	1.21 (0.94 – 1.54)
p-value for interaction between education and allostatic load for all men	0.02	0.37	0.23
**Non-Hispanic Black**			
College graduate, Low AL	1.00 (Referent)	1.00 (Referent)	1.00 (Referent)
College graduate, High AL	1.42 (0.66–3.07)	0.92 (0.43–2.00)	0.89 (0.41 – 1.92)
Some college, Low AL	1.05 (0.51–2.15)	1.46 (0.72–2.98)	1.30 (0.63 – 2.69)
Some college, High AL	1.58 (0.81–3.11)	1.16 (0.59–2.28)	1.00 (0.50 – 2.00)
HS or GED, Low AL	0.86 (0.43–1.72)	1.31 (0.66–2.61)	1.06 (0.53 – 2.15)
HS or GED, High AL	1.63 (0.85–3.12)	1.26 (0.66–2.45)	0.97 (0.50 – 1.90)
Less than HS, Low AL	1.08 (0.55–2.12)	1.30 (0.66–2.55)	0.91 (0.45 – 1.83)
Less than HS, High AL	3.30 (1.79 – 6.11)	1.44 (0.77–2.69)	0.97 (0.51 – 1.86)
p-value for interaction between education and allostatic load for non-Hispanic Black men	0.13	0.89	0.89
**Non-Hispanic White**			
College graduate, Low AL	1.00 (Referent)	1.00 (Referent)	1.00 (Referent)
College graduate, High AL	1.73 (1.26–2.38)	1.12 (0.81–1.55)	0.99 (0.71 – 1.37)
Some college, Low AL	0.96 (0.68–1.35)	1.11 (0.79–1.57)	1.01 (0.72 – 1.44)
Some college, High AL	2.20 (1.62–2.98)	1.54 (1.13–2.10)	1.30 (0.95 – 1.79)
HS or GED, Low AL	1.01 (0.73–1.34)	1.19 (0.86–1.64)	1.02 (0.74 – 1.42)
HS or GED, High AL	2.63 (1.99–3.47)	1.77 (1.33–2.35)	1.44 (1.07 – 1.94)
Less than HS, Low AL	1.70 (1.23–2.34)	1.32 (0.95–1.83)	1.08 (0.76 – 1.52)
Less than HS, High AL	3.23 (2.46–4.25)	1.64 (1.23–2.20)	1.27 (0.93 – 1.74)
p-value for interaction between education and allostatic load for non-Hispanic White men	0.32	0.74	0.57
**Among Latino Men**			
College graduate, Low AL	1.00 (Referent)	1.00 (Referent)	1.00 (Referent)
College graduate, High AL	0.69 (0.22–2.21)	0.43 (0.13–1.37)	0.36 (0.11 – 1.21)
Some college, Low AL	0.42 (0.17–1.05)	0.65 (0.26–1.61)	0.55 (0.23 – 1.34)
Some college, High AL	1.01 (0.45–2.28)	0.80 (0.35–1.81)	0.67 (0.30 – 1.52)
HS or GED, Low AL	0.22 (0.09–0.56)	0.42 (0.17–1.06)	0.38 (0.15 – 0.94)
HS or GED, High AL	1.29 (0.62–2.66)	1.03 (0.50–2.15)	0.86 (0.42 – 1.77)
Less than HS, Low AL	0.75 (0.34–1.46)	0.85 (0.43–1.65)	0.67 (0.34 – 1.35)
Less than HS, High AL	2.02 (1.06–3.85)	0.99 (0.51–1.91)	0.77 (0.39 – 1.52)
p-value for interaction between education and allostatic load for Latino men	<0.01	<0.01	<0.01
**Other Men**			
College graduate, Low AL	1.00 (Referent)	1.00 (Referent)	1.00 (Referent)
College graduate, High AL	0.86 (0.08–9.44)	0.59 (0.05–6.77)	0.46 (0.04 – 5.04)
Some college, Low AL	## (##)	## (##)	## (##)
Some college, High AL	3.45 (0.58–20.57)	2.46 (0.40–15.11)	2.17 (0.40 – 11.82)
HS or GED, Low AL	0.91 (0.13–6.42)	1.22 (0.18–8.37)	1.13 (0.17 – 7.65)
HS or GED, High AL	4.59 (0.91–23.28)	3.28 (0.63–17.15)	2.04 (0.42 – 9.79)
Less than HS, Low AL	1.99 (0.37–10.71)	2.05 (0.38–10.97)	1.55 (0.31 – 7.81)
Less than HS, High AL	3.01 (0.62–14.68)	1.78 (0.32–9.92)	1.12 (0.19 – 6.49)
p-value for interaction between education and allostatic load for Other men	<0.01	<0.01	<0.01

[a]The sample included 20,529 NHANES men participants with 1,501 cancer-related deaths and 4,638 all-cause deaths.
[b]Competing risk analysis is estimated using the Fine–Gray proportional hazard models with the sample treated as a simple random sample. [c]Survey-weighted Cox proportional hazards model.
[d]Fully adjusted for age, family poverty to income ratio, current smoker status, cancer, time-period, congestive heart failure, and heart attack. ## represents undefined estimates due to small sample sizes.

© 2024 Li C et al. *JAMA Network Open.*

eTable 3. Risk of cancer death associated with educational attainment (dichotomous) and allostatic load status low/high groups and stratified by race. Among 20,529 men surveyed by NHANES years 1988–2010. Follow up data through December 31, 2019.

Educational Attainment and Allostatic Load (AL) Status	No. at Risk Un-weighted[a]	No. Cancer Deaths (Weighted %)[b]	Mean Survival Months (SE)[c]	Hazard Ratio[d] (95% CI)		
				Unadjusted	Age-Adjusted	Fully Adjusted[d]
**Among All Men**						
High Education, Low AL	4403	186 (3.23)	333.8 (0.49)	1.00 (Referent)	1.00 (Referent)	1.00 (Referent)
Low Education, Low AL	6827	310 (3.92)	364.0 (0.47)	1.29 (0.98 – 1.70)	1.38 (1.07-1.77)	1.09 (0.85 – 1.39)
High Education, High AL	3145	253 (6.14)	310.1 (0.80)	2.24 (1.77 – 2.83)	1.16 (0.91-1.47)	1.09 (0.85 – 1.39)
Low Education, High AL	6084	742 (11.06)	326.1 (0.78)	4.21 (3.39 – 5.30)	2.11 (1.67-2.65)	1.65 (1.31 – 2.08)
p-value for interaction between education and allostatic load for all men:				0.03	0.07	0.02
**Among White Men**						
High Education, Low AL	2756	130 (3.14)	281.4 (0.7)	1.00 (Referent)	1.00 (Referent)	1.00 (Referent)
Low Education, Low AL	2467	156 (4.63)	301.8 (1.1)	1.43 (1.03-2.00)	1.42 (1.05-1.93)	1.10 (0.81-1.48)
High Education, High AL	1857	178 (6.74)	290.3 (1.6)	2.32 (1.80-2.99)	1.21 (0.94-1.56)	1.12 (0.87-1.44)
Low Education, High AL	2456	355 (12.63)	273.8 (2.0)	4.68 (3.73-5.89)	2.27 (1.79-2.89)	1.79 (1.39-2.30)
p-value for interaction between education and allostatic load for White men:				0.09	0.12	0.02
**Among Black Men**						
High Education, Low AL	693	35 (3.86)	271.8 (1.5)	1.00 (Referent)	1.00 (Referent)	1.00 (Referent)
Low Education, Low AL	1391	68 (4.16)	285.2 (1.1)	1.05 (0.65-1.70)	1.05 (0.68-1.63)	0.79 (0.49-1.29)
High Education, High AL	717	53 (5.75)	246.4 (1.7)	1.57 (0.91-2.71)	0.75 (0.44-1.28)	0.76 (0.44-1.30)
Low Education, High AL	1595	212 (10.04)	304.8 (2.3)	2.79 (1.74-4.45)	1.09 (0.69-1.72)	0.84 (0.51-1.36)
p-value for interaction between education and allostatic load for Black men:				0.07	0.24	0.26
**Among Latino Men**						
High Education, Low AL	704	19 (2.19)	333.2 (1.8)	1.00 (Referent)	1.00 (Referent)	1.00 (Referent)
Low Education, Low AL	2729	80 (1.54)	344.5 (0.7)	0.66 (0.37-1.18)	0.73 (0.41-1.28)	0.65 (0.36-1.17)
High Education, High AL	443	18 (1.81)	292.4 (2.0)	0.92 (0.43-1.98)	0.50 (0.23-1.09)	0.54 (0.25-1.18)
Low Education, High AL	1860	164 (5.25)	323.1 (1.8)	2.47 (1.28-4.75)	1.00 (0.62-1.93)	0.90 (0.41-1.97)
p-value for interaction between education and allostatic load for Latino men:				<0.01	0.02	0.01
**Among Other Men**						
High Education, Low AL	250	2 (0.83)	39.0 (##)	1.00 (Referent)	1.00 (Referent)	1.00 (Referent)
Low Education, Low AL	240	1 (3.16)	232.8 (1.5)	3.25 (0.53-19.77)	3.03 (0.50-18.33)	3.20 (0.67-15.26)
High Education, High AL	128	4 (2.52)	120.6 (1.2)	3.43 (0.46-25.73)	1.67 (0.21-12.97)	1.91 (0.26-14.18)
Low Education, High AL	173	11 (8.27)	296.3 (4.5)	8.96 (1.44-55.68)	4.51(0.63-32.06)	2.74 (0.44-17.29)
p-value for interaction between education and allostatic load for other men:				0.86	0.93	0.51

[a]The sample included 20,529 NHANES men participants with 1,501 cancer-related deaths.
[b]Percentages are weighted. Cox proportional hazard models are estimated using NHANES survey weighting. Weighted population *N* = 84,363,491 men and *n* = 4,657,724 cancer-related deaths.
[c]Mean survival months are unweighted.
[d]Survey-weighted Cox proportional hazards model.
[e]Fully adjusted is for age, family poverty to income ratio, current smoker status, cancer, time-period, congestive heart failure, and heart attack.
# indicates null result due to small number in analysis. High education are participants with some college and college graduate or above. Low education are participants with less than a high school (HS) education, high school graduate, general education development test (GED), or equivalent.

© 2024 Li C et al. *JAMA Network Open.*

eTable 4. Association between allostatic load status and risk of cancer death, stratified by educational attainment (dichotomous) and race. Among 20,529 men surveyed by NHANES years 1988 – 2010. Follow up data through December 31, 2019.

Educational Attainment and Allostatic Load (AL) Status	No. Cancer Deaths[a] (Weighted %)[b]	Hazard Ratio[c] (95% CI), *p*-value		
		Unadjusted	Age-Adjusted	Fully Adjusted[d]
Non-Hispanic Black				
High Education, Low AL	35 (3.86)	1.00 (Referent)	1.00 (Referent)	1.00 (Referent)
High Education, High AL	53 (5.75)	1.56 (0.90–2.69)	0.81 (0.35–1.92)	0.75 (0.44 – 1.27)
Low Education, Low AL	68 (4.16)	1.00 (Referent)	1.00 (Referent)	1.00 (Referent)
Low Education, High AL	212 (10.04)	2.66 (1.93-3.68)	1.02 (0.75–1.40)	1.04 (0.74 – 1.45)
Non-Hispanic White				
High Education, Low AL	130 (3.14)	1.00 (Referent)	1.00 (Referent)	1.00 (Referent)
High Education, High AL	178 (6.74)	2.33 (1.80-3.00)	0.92 (0.65-1.31)	1.09 (0.84 – 1.42)
Low Education, Low AL	156 (4.63)	1.00 (Referent)	1.00 (Referent)	1.00 (Referent)
Low Education, High AL	355 (12.63)	3.27 (2.43-4.40)	1.66 (1.25-2.20)	1.67 (1.26 – 2.20)
Latino/ Other Hispanic				
High Education, Low AL	19 (2.19)	1.00 (Referent)	1.00 (Referent)	1.00 (Referent)
High Education, High AL	18 (1.81)	0.93 (0.44-1.96)	0.33 (0.10-1.21)	0.49 (0.23 – 1.06)
Low Education, Low AL	80 (1.54)	1.00 (Referent)	1.00 (Referent)	1.00 (Referent)
Low Education, High AL	164 (5.25)	3.77 (2.33-6.10)	1.46 (0.85-2.51)	1.46 (0.80 – 2.67)
Other/Mixed Race				
High Education, Low AL	2 (0.83)	1.00 (Referent)	1.00 (Referent)	1.00 (Referent)
High Education, High AL	4 (2.52)	3.29 (0.43-25.22)	1.04 (0.14 – 7.64)	1.60 (0.49 – 5.17)
Low Education, Low AL	1 (3.16)	1.00 (Referent)	1.00 (Referent)	1.00 (Referent)
Low Education, High AL	11 (8.27)	2.76 (0.81-9.33)	1.61 (0.44-5.84)	0.97 (0.27 – 3.44)

[a]The sample included 20,529 NHANES men participants with 1,501 cancer-related deaths.
[b]Percentages are weighted. Hazard Ratios are estimated using Cox proportional hazard regression. Weighted population N = 84,363,491 men and n = 4,657,724 cancer-related deaths.
[c]Survey-weighted Cox proportional hazards model.
[d]Fully adjusted is for age, family poverty to income income ratio, current smoker status, cancer, time-period, congestive heart failure, and heart attack.
High education are participants with some college and college graduate or above. Low education are participants with less than a high school (HS) education, high school graduate, general education development test (GED), or equivalent.

© 2024 Li C et al. *JAMA Network Open.*

eTable 5. Unweighted analysis (treated as simple random sample) of risk of cancer death associated with educational attainment and allostatic load status and stratified by race. Among 20,529 men surveyed by NHANES years 1988 – 2010. Follow up data through December 31, 2019

Educational Attainment and Allostatic Load (AL) Status	No. at Risk Unweighted[a]	No. Cancer Deaths (Unweighted %)[b]	Mean Survival Months (SE)[c]	Hazard Ratio[d] (95% CI)		
				Unadjusted	Age-Adjusted	Fully Adjusted[d]
**Among All Men**						
College graduate, Low AL	2065	94 (4.6)	281.9 (0.81)	1.00 (Referent)	1.00 (Referent)	1.00 (Referent)
College graduate, High AL	1350	101 (7.5)	264.6 (1.28)	1.76 (1.33 – 2.34)	1.01 (0.77 – 1.35)	0.91 (0.69 – 1.21)
Some college, Low AL	2338	92 (3.9)	331.7 (1.07)	0.85 (0.64 – 1.13)	1.28 (0.96 – 1.71)	1.13 (0.84 – 1.51)
Some college, High AL	1795	152 (8.5)	292.4 (1.54)	2.10 (1.62 – 2.71)	1.50 (1.16 – 1.95)	1.26 (0.97 – 1.63)
HS or GED, Low AL	3129	118 (3.8)	338.3 (0.78)	0.78 (0.59 – 1.02)	1.28 (0.98 – 1.68)	1.06 (0.80 – 1.40)
HS or GED, High AL	2385	251 (10.5)	290.4 (1.49)	2.41 (1.90 – 3.06)	1.82 (1.44 – 2.31)	1.44 (1.12 – 1.84)
Less than HS, Low AL	3698	192 (5.2)	338.2 (0.95)	1.08 (0.84 – 1.38)	1.36 (1.06 – 1.74)	1.03 (0.80 – 1.34)
Less than HS, High AL	3699	491 (13.3)	307.1 (1.74)	3.51 (1.63 – 6.00)	1.84 (1.47 – 2.30)	1.38 (1.08 – 1.76)
p-value for interaction between education and allostatic load for all men:				<0.01	0.35	0.16
**Among White Men**						
College graduate, Low AL	1468	71 (4.8)	282.3 (0.9)	1.00 (Referent)	1.00 (Referent)	1.00 (Referent)
College graduate, High AL	901	80 (8.9)	263.4 (1.7)	1.97 (1.43 – 2.72)	1.11 (0.80 – 1.53)	0.99 (0.71 – 1.37)
Some college, Low AL	1288	59 (4.6)	280.3 (1.2)	0.97 (0.69 – 1.37)	1.32 (0.93 – 1.86)	1.13 (0.80 – 1.60)
Some college, High AL	956	98 (10.3)	287.1 (2.5)	2.65 (1.95 – 3.60)	1.74 (1.28 – 2.36)	1.39 (1.01 – 1.89)
HS or GED, Low AL	1534	77 (5.0)	276.8 (0.9)	1.03 (0.74 – 1.42)	1.37 (0.99 – 1.89)	1.09 (0.78 – 1.52)
HS or GED, High AL	1232	162 (13.2)	272.6 (2.3)	3.25 (2.46 – 4.30)	2.09 (1.58 – 2.77)	1.63 (1.21 – 2.18)
Less than HS, Low AL	933	79 (8.5)	294.9 (2.3)	1.98 (1.44 – 2.73)	1.57 (1.14 – 2.17)	1.12 (0.80 – 1.57)
Less than HS, High AL	1224	193 (15.8)	265.3 (3.2)	4.95 (3.76 – 6.50)	2.13 (1.61 – 2.82)	1.56 (1.15 – 2.11)
p-value for interaction between education and allostatic load for White men:				0.28	0.58	0.31
**Among Black Men**						
College graduate, Low AL	229	11 ()	189.5 (1.8)	1.00 (Referent)	1.00 (Referent)	1.00 (Referent)
College graduate, High AL	232	16 (6.9)	224.9 (2.4)	1.55 (0.72 – 3.33)	0.93 (0.43 – 2.02)	0.97 (0.45 – 2.11)
Some college, Low AL	464	24 (5.2)	272.4 (1.7)	1.06 (0.52 – 2.15)	1.51 (0.74 – 2.08)	1.42 (0.69 – 2.90)
Some college, High AL	485	37 (7.6)	246.2 (2.1)	1.71 (0.87 – 3.34)	1.04 (0.53 – 2.04)	0.97 (0.49 – 1.91)
HS or GED, Low AL	701	31 (4.4)	286.9 (1.3)	0.86 (0.43 – 1.71)	1.36 (0.69 – 2.71)	1.13 (0.56 – 2.26)
HS or GED, High AL	652	57 (8.7)	290.3 (2.3)	1.75 (0.92 – 3.34)	1.25 (0.65 – 2.39)	1.01 (0.52 – 1.94)
Less than HS, Low AL	690	37 (5.4)	246.6 (1.4)	1.13 (0.57 – 2.21)	1.42 (0.72 – 2.78)	0.98 (0.49 – 1.95)
Less than HS, High AL	943	155 (16.4)	294.2 (3.5)	4.36 (2.37 – 8.04)	1.72 (0.93 – 3.19)	1.21 (0.64 – 2.29)
p-value for interaction between education and allostatic load for Black men:				0.03	0.49	0.41
**Among Latino Men**						
College graduate, Low AL	228	10 (4.4)	200.1 (1.5)	1.00 (Referent)	1.00 (Referent)	1.00 (Referent)
College graduate, High AL	141	4 (2.8)	202.5 (2.2)	0.78 (0.25 – 2.49)	0.48 (0.15 – 1.55)	0.39 (0.12 – 1.25)
Some college, Low AL	476	9 (1.9)	334.6 (2.0)	0.42 (0.17 – 1.03)	0.70 (0.28 – 1.73)	0.55 (0.22 – 1.39)
Some college, High AL	302	14 (4.6)	292.3 (2.4)	1.10 (0.49 – 2.47)	0.86 (0.38 – 1.94)	0.73 (0.32 – 1.66)
HS or GED, Low AL	766	8 (1.0)	344.6 (0.3)	0.22 (0.09 – 0.56)	0.46 (0.18 – 1.16)	0.43 (0.17 – 1.10)
HS or GED, High AL	434	27 (6.2)	301.4 (2.4)	1.36 (0.66 – 2.81)	1.04 (0.50 – 2.15)	0.90 (0.43 – 1.89)
Less than HS, Low AL	1963	72 (3.7)	342.6 (1.0)	0.76 (0.39 – 1.46)	0.91 (0.47 – 1.78)	0.75 (0.37 – 1.51)
Less than HS, High AL	1426	137 (9.6)	320.4 (2.2)	2.38 (1.25 – 4.52)	1.06 (0.55 – 2.03)	0.85 (0.42 – 1.69)
p-value for interaction between education and allostatic load for Latino men:				0.07	0.29	0.23
**Among Other Men**						
College graduate, Low AL	140	2 (1.4)	39.0 (##)	1.00 (Referent)	1.00 (Referent)	1.00 (Referent)
College graduate, High AL	76	1 (1.3)	122.0 (##)	0.89 (0.08 – 9.83)	0.50 (0.05 – 5.61)	0.48 (0.04 – 5.55)

© 2024 Li C et al. *JAMA Network Open.*

Some college, Low AL	110	0 (0.0)	## (##)	## (##)	## (##)	## (##)
Some college, High AL	52	3 (5.8)	65.8 (1.6)	3.99 (0.67 – 23.91)	2.35 (0.38 – 14.55)	2.52 (0.36 – 17.80)
HS or GED, Low AL	128	2 (1.6)	232.8 (2.2)	0.93 (0.13 – 6.59)	1.37 (0.19 – 9.81)	1.46 (0.18 – 11.67)
HS or GED, High AL	67	5 (7.5)	291.6 (8.8)	4.93 (0.96 – 25.43)	3.22 (0.62 – 16.86)	2.66 (0.45 – 15.67)
Less than HS, Low AL	112	4 (3.6)	188.4 (1.4)	2.04 (0.37 – 11.15)	2.02 (0.37 – 11.12)	1.90 (0.30 – 12.19)
Less than HS, High AL	106	6 (5.7)	231.8 (3.3)	3.48 (0.70 – 17.30)	1.76 (0.34 – 9.12)	1.34 (0.22 – 8.29)
p-value for interaction between education and allostatic load for other men:				0.77	0.85	0.90

[a]The sample included 20,529 NHANES men participants with 1,501 cancer-related deaths.
[b]Percentages are unweighted.
[c]Mean survival months are unweighted.
[d]Unweighted Cox proportional hazards model.
[e]Fully adjusted is for age, family poverty to income ratio, current smoker status, cancer, time-period, congestive heart failure, and heart attack.
indicates null result due to small number in analysis.

© 2024 Li C et al. *JAMA Network Open.*
