# Supplementary material for: Allostatic Load, Educational Attainment, and Risk of Cancer Mortality Among US Men
Source: JAMA Netw Open. 2024 Dec 10;7(12):e2449855. doi: 10.1001/jamanetworkopen.2024.49855 (PMC11632542; doi:10.1001/jamanetworkopen.2024.49855)
Supplement: Supplement 2. — Data Sharing Statement [file jamanetwopen-e2449855-s002.pdf]

Data Sharing Statement

Li. Allostatic Load, Educational Attainment, and Risk of Cancer Mortality Among US Men.
JAMA Netw Open. Published December 10, 2024. doi:10.1001/jamanetworkopen.2024.49855

Data
Data available: Yes
Data types: Deidentified participant data
How to access data: jx.moore@uky.edu
When available: With publication

Supporting Documents
Document types: Statistical/analytic code
How to access documents: jx.moore@uky.edu
When available: With publication

Additional Information
Who can access the data: anyone requesting the data
Types of analyses: For research purposes.
Mechanisms of data availability: Without investigator support
